# Supplementary material for: Metastable oscillatory modes emerge from synchronization in the brain spacetime connectome
Source: Commun Phys. Author manuscript; Available in PMC 2024 Jan 29. (PMC7615562; doi:10.1038/s42005-022-00950-y)
Supplement: Supp 3 [file EMS189542-supplement-Supp_3.docx]

Description of Additional Supplementary Files

**File name:** Supplementary Movie 1

**Description:** Metastable Oscillatory Modes (MOMs) emerge transiently from interactions in the Connectome spacetime structure only for sufficient coupling and conduction times. Each brain area is represented as a sphere located at its centre of gravity. A colour code is used to highlight the brain areas with power exceeding 5 standard deviations from the baseline power at a given time point. While the structural connectome is the same for all simulations, MOMs only emerge at reduced frequencies in the presence of Conduction Delays 〈τ〉 and for sufficient Coupling strength (K).
