# Supplementary material for: Metastable oscillatory modes emerge from synchronization in the brain spacetime connectome
Source: Commun Phys. Author manuscript; Available in PMC 2024 Jan 29. (PMC7615562; doi:10.1038/s42005-022-00950-y)
Supplement: Supp 1 [file EMS189542-supplement-Supp_1.pdf]

nature portfolio

Peer Review File

Metastable oscillatory modes emerge from synchronization in the brain spacetime connectome

Open Access This file is licensed under a Creative Commons Attribution 4.0 International License, which permits use, sharing, adaptation, distribution and reproduction in any medium or format, as long as you give appropriate credit to the original author(s) and the source, provide a link to the Creative Commons license, and indicate if changes were made. In the cases where the authors are anonymous, such as is the case for the reports of anonymous peer reviewers, author attribution should be to 'Anonymous Referee' followed by a clear attribution to the source work. The images or other third party material in this file are included in the article's Creative Commons license, unless indicated otherwise in a credit line to the material. If material is not included in the article's Creative Commons license and your intended use is not permitted by statutory regulation or exceeds the permitted use, you will need to obtain permission directly from the copyright holder. To view a copy of this license, visit http://creativecommons.org/licenses/by/4.0/.

Reviewers' comments:

Reviewer #1 (Remarks to the Author):

In the manuscript entitled "Synchronization in the connectome: Metastable oscillatory modes emerge from interactions in the brain spacetime network," the authors replicated metastable oscillatory modes empirically observed with MEG/EEG by simulating interactions between oscillator models on the structural connectome.

This paper is well written and organized, but I have some difficulty in understanding which part is novel for the field. Similar findings are already published by the same first author in 2014 (Cabral et al., NeuroImage 90, 423-435). The authors cited this paper in Introduction and said
1. "While these numerical results reveal the critical role of time delays to generate collective oscillations at reduced frequencies, the underlying mechanistic principle remains unclear"
and
2. "Further, it remains to be verified whether this phenomenon holds in the more realistic setting, wherein local oscillations have fluctuating amplitude, as observed empirically in electrophysiological recordings of neural activity."

My major comments are as follows:

1. Could the authors explicitly specify what underlying mechanistic principle (that remained unclear in Cabral et al., 2014) is now clarified in this paper?

2. The oscillator model used in Cabral et al. (2014) was Kuramoto and that in this paper is Stuart-Landau. As shown in SI, the Kuramoto model is a reduced version of the Stuart-Landau model. The Stuart-Landau can replicate properties that the simpler Kuramoto can also replicate is somehow trivial. Could the author clarify the real gain of using the Stuart-Landau model in this paper?

Minor:
P18 L257 in SI, what are the state-of-the-art tractography algorithms? Please identify which tractography method was used to make the connectome in this study.

Reviewer #2 (Remarks to the Author):

The manuscript "Synchronization in the connectome: Metastable oscillatory modes emerge from interactions in the brain spacetime network" by Cabral et al. proposes a connection between transient brain rhythms and weekly stable synchronization of distant brain areas, emphasizing the role of signal transmission delays in the emergence of transient rhythms. To support their hypothesis, the authors present results of extensive numerical simulations carried out on a network of 90 Stuart-Landau oscillators, representing 90 brain areas, where connection strengths and conduction delays are derived from physical brain connectivity data.

The construction of the network based on real data is in itself a nice piece of work. Moreover, the simulations are extensive and contain rich results, some of which are already spelled out in the manuscript. The main findings indicate that conduction delays play a crucial role in shaping the frequency spectrum of coupled systems, and that synchronization under delays induces a shift to lower frequencies as well as a decrease in amplitude. Some of the observations are probably not completely surprising for theoretical scientists working with delay systems (as already referenced in the manuscript), but they are demonstrated here more concretely and quantitatively for a more realistic brain-like structure. It can be expected that the results provided here can provide even further inspiration for future studies in unraveling the details of brain oscillations, as well as in the wider area of complex networks.

The manuscript is generally well-written, although a thorough reading and editing is recommended. There are some spelling errors (e.g. "condtuction") and some consistency in typesetting notation (e.g. consider how differently the function "sin" is formatted in equations (3) and (4)). Finally I am not sure why the symbol "*" is used for multiplication in equations; it is not very standard (except in coding) and it is also not used consistently throughout the manuscript.

Reviewer #3 (Remarks to the Author):

The authors use a brain network model to simulate brain dynamics and study spatio-temporal synchronization dynamics of brain regions. They break down oscillations into standard frequency bands studied in neuroscience and compare simulated results to that obtained from MEG data. They show that for certain ranges of regional delays and coupling strengths that the model can reproduce the slow frequency brain observations observed in real data. I fully support the use of brain network models to explore oscillations and synchrony in brain dynamics, but I feel that there is much analysis missing from the paper and that the main results are somewhat expected. This paper feels like a starting point for a more rich analysis that could link more directly to questions in neuroscience.

Major general concerns:

1. It appears that the authors are building their structural matrix from the average connectome calculated from 32 individuals and then averaged over individuals. It has been shown that individual variability in the structural network used in these models leads to enhanced variability in the dynamics that they output. If the goal is to compare with individual MEG data, the structural networks used in the model need to be the structural networks from those same individuals. From what I can tell, no attempt was made to model separate dynamics at the individual level, and given that the MEG data is from 89 subjects, it is unclear if there is any overlap between the subjects used to generate structural and functional networks.

2. Given the above, I don't understand the motivation to even attempt to look at how the model dynamics align with individual MEG power spectrums. I wouldn't expect the model to match well with across subjects given that the underlying structural matrix isn't subject-specific.

3. The authors discuss spatial synchronization, but seem to be looking only at things such as the number of units recruited into MOMs and the frequency specific envelop covariance matrices. For this to be interesting to a neuroscientist, one wants to know which brain regions are recruited into the oscillations. Do those regions relate to known cognitive or functional communities? One expects there to be metastable communities that synchronize at different frequency bands. Which brain regions are involved in these patterns is the interesting part.

More specific comments:

1. The analysis was performed on the 90 region AAL atlas. Do the results hold for other parcellations with a larger number of regions?

2. I understand the desire to look at the dependance on coupling strength and delays, but given that much other work looking at coupled oscillators outside of neuroscience applications also has investigated this, an interesting and relevant parameter to explore in this case would be the natural frequency of the oscillators. From what I can tell, all oscillators were set to a natural frequency of 40 Hz. Are the results robust to different choices? I would expect that different brain regions oscillate at different natural frequencies, so exploring how the model depends on this parameter choice could be a large contribution to linking coupled oscillator theory to brain dynamics.

3. Line 260-262: “Importantly we note that for the model to exhibit the predicted behavior, the integration step for numerical simulations needs to be sufficiently small…”. This is a concerning statement. Of course one much choose an integration step small enough that the results are stable – this is just related to correctly performing numerical integration. If the results are dependent upon the integration time step, then the numerical integration is not being performed correctly. Since the authors are injecting noise into the equations, it might be necessary to perform stochastic numerical integration – although it is unclear if this was done.

<u>**COMMSPHYS-21-0962-T**</u>

Title: Synchronization in the connectome: Metastable oscillatory modes emerge from interactions in the brain spacetime network

Authors: Joana Cabral, Francesca Castaldo, Jakub Vohryzek, Vladimir Litvak, Christian Bick, Renaud Lambiotte, Karl Friston, Morten L. Kringelbach and Gustavo Deco

<u>Response to Reviewers</u>

We would like to thank the reviewers for their insightful and constructive comments, which have certainly strengthened our manuscript. We have carefully addressed each of the reviewers' concerns, commentaries and suggestions. As you can see below, we have performed additional simulations and analyses including structural connectomes from a different dataset and in a different parcellation, reducing the integration step by 2 orders of magnitude and adding a spread in the natural frequencies, which resulted in a new section in the manuscript and eight new supplementary figures. These substantial analyses and major revisions to the text have confirmed the robustness and generalizability of the results presented herein and helped clarify the novelty and relevance of this work. We trust that these changes and clarifications meet the exacting standards expected for publication in Communications Physics.

Reviewer #1:

In the manuscript entitled "Synchronization in the connectome: Metastable oscillatory modes emerge from interactions in the brain spacetime network," the authors replicated metastable oscillatory modes empirically observed with MEG/EEG by simulating interactions between oscillator models on the structural connectome.

This paper is well written and organized, but I have some difficulty in understanding which part is novel for the field. Similar findings are already published by the same first author in 2014 (Cabral et al., NeuroImage 90, 423-435).

Authors' reply: Thank you for raising the crucial point of novelty, and we appreciate the opportunity to explain more clearly the advances with respect to previous studies and the relevance of this work for the neuroscience field. Please find below our detailed response to the reviewer's comments and a copy of the new text insertions in the Abstract, Introduction, Methods and Discussion. All changes in the manuscript are marked in blue font.

The authors cited this paper in Introduction and said
1. "While these numerical results reveal the critical role of time delays to generate collective oscillations at reduced frequencies, the underlying mechanistic principle remains unclear"

2. "Further, it remains to be verified whether this phenomenon holds in the more realistic setting, wherein local oscillations have fluctuating amplitude, as observed empirically in electrophysiological recordings of neural activity."

My major comments are as follows:

1. Could the authors explicitly specify what underlying mechanistic principle (that remained unclear in Cabral et al., 2014) is now clarified in this paper?

Authors' reply: We agree that the main motivation of this work was not sufficiently explicit, and we do now elaborate on this subject. Overall, this work was designed to improve the construct validity of the mechanistic theory proposed in Cabral et al., 2014 for the emergence of collective oscillations in brain activity.

The reviewer is correct that the mechanistic principle is the same proposed in Cabral et al., 2014 (and we have rephrased the text accordingly) and we now focus on the true novelty, which lies in:

1) The analytic prediction of the synchronization frequency in the connectome structure, which was not addressed in Cabral et al. (2014). Here, we show that the global peak frequency in simulations can be analytically predicted by the same equation used in Niebur et al. (1991) to predict the collective frequency in a regular lattice of coupled limit-cycle oscillators with nearest-neighbour coupling.

2) The extension from phase oscillators (with constant amplitude) to Stuart Landau oscillators (with amplitude modulation) to improve the biophysical realism (we explain the importance of this issue for neuroscience in the reply to the following comment).

3) The verification that the mechanism extends to coupled oscillators that are *not* in the limit cycle regime, while the work from Niebur et al. (1991) only referred to limit cycle oscillators.

4) The definition of metrics to characterise emergent collective oscillations, which we term 'metastable oscillatory modes' (MOMs). In Cabral et al (2014) only the envelope correlation matrices in each frequency band were reported. Here, we define MOMs as groups of nodes that simultaneously increase the power at a common collective frequency, and characterise them across frequency bands in terms of number of units, duration and occupancy. We show that MOM properties vary strongly as a function of global parameters (the global coupling strength and the average time delay), while the structural network remains unchanged.

Niebur, E., Schuster, H. G., & Kammen, D. M. (1991). Collective frequencies and metastability in networks of limit-cycle oscillators with time delay. *Physical review letters*, *67*(20), 2753.

2. The oscillator model used in Cabral et al. (2014) was Kuramoto and that in this paper is Stuart-Landau. As shown in SI, the Kuramoto model is a reduced version of the Stuart-Landau model. The Stuart-Landau can replicate properties that the simpler Kuramoto can also replicate is somehow trivial. Could the author clarify the real gain of using the Stuart-Landau model in this paper?

Authors' reply: Thank you for raising the importance of clarifying the benefit of using a model of coupled Stuart-Landau (SL) oscillators instead of the Kuramoto model of coupled phase oscillators (we note that the Kuramoto model represents a system of coupled units, whereas the SL equation represents the unit itself). It is true that the SL equation can be reduced to a phase oscillator, but this

reduction does not take into account the amplitude dynamics (Ashwin et al., 2016). This reduction is usually accepted when the SL oscillators are in the limit-cycle regime , that is, on the supercritical side of the bifurcation (i.e., when the parameter ‘a’ is positive). If instead the SL oscillators are in the subcritical regime, they exhibit only input-driven damped oscillations, and the reduction to a phase oscillator cannot adequately capture the full dynamics of the system since it completely neglects the amplitude dynamics.

This is important for the neuroscience field, since empirical electrophysiological recordings show that local field oscillations in the gamma frequency band are *not* limit-cycle oscillations, but instead emerge only transiently, lasting from hundreds of milliseconds to a few seconds (Singer, 1993; Buzsáki et al., 2012). Therefore, the substantial reduction of local field potentials to phase oscillators in Cabral et al. (2014) has raised a critical debate on the generalizability of the proposed mechanism to more realistic settings, given the demonstrated importance of considering the amplitude dynamics on the connectivity between phases (Daffertshofer and van Wijk, 2011, Daffersthofer et al, 2018, Siems et al., 2020). As such, demonstrating that the mechanism extends to ‘damped’ oscillators is crucial to improve the biophysical realism of this mechanistic theory.

Ashwin, P., Coombes, S., & Nicks, R. (2016). Mathematical Frameworks for Oscillatory Network Dynamics in Neuroscience. The Journal of Mathematical Neuroscience, 6(1), 2. doi:10.1186/s13408-015-0033-6

Buzsáki, G., Anastassiou, C. A. & Koch, C. The origin of extracellular fields and currents—EEG, ECoG, LFP and spikes. Nature reviews neuroscience 13, 407 (2012).

Daffertshofer, A., & van Wijk, B. (2011). On the influence of amplitude on the connectivity between phases. *Frontiers in neuroinformatics*, *5*, 6.

Daffertshofer, A., Ton, R., Kringelbach, M. L., Woolrich, M., & Deco, G. (2018). Distinct criticality of phase and amplitude dynamics in the resting brain. *Neuroimage*, *180*, 442-447.

Siems, M., & Siegel, M. (2020). Dissociated neuronal phase-and amplitude-coupling patterns in the human brain. *NeuroImage*, *209*, 116538.

Singer, W. (1993). Synchronization of cortical activity and its putative role in information processing and learning. *Annual review of physiology*, *55*(1), 349-374.

Text modified in the Abstract:

“However, the principles underwriting coherent oscillations and their link with neural activity remain under debate. Here, we revisit the mechanistic hypothesis that transient brain rhythms are a signature of metastable synchronization, occurring at reduced collective frequencies due to delays between brain areas. We consider a system of damped oscillators – approximating the short-lived gamma-frequency oscillations generated within neuronal circuits – coupled according to the diffusion weighted tractography between brain areas. Varying only the global coupling strength and conduction speed, we identify a critical regime where spatially and spectrally resolved metastable oscillatory modes (MOMs) emerge at sub-gamma frequencies, approximating the MEG power spectra from 89 healthy individuals at rest. Further, we demonstrate that the frequency, duration,

and scale of MOMs – as well as the frequency-specific envelope functional connectivity – can be controlled by global model parameters, while the connectome structure remains unchanged. Grounded in the physics of delay-coupled oscillators, these numerical analyses demonstrate how interactions between locally generated fast oscillations in the connectome spacetime structure can lead to the emergence of collective brain rhythms organized in space and time."

Text modified in the Introduction:

'[...] it remains to be verified whether this phenomenon holds in the more realistic setting, wherein local oscillations have fluctuating amplitude – which is neglected in the Kuramoto model –, as observed empirically in electrophysiological recordings of neural activity [45,46]. Furthermore, understanding the parameters that control the duration, size and occupancy of collective oscillations is crucial to inform the prediction of therapeutic strategies aimed at modulating dysfunctional oscillatory brain activity.

To address these fundamental questions, we build a phenomenological brain network model with realistic connectivity and time delays, where each node is described by a Stuart-Landau oscillator operating in the subcritical regime, i.e., responding to a stimulus with an oscillation with decaying amplitude [34,35,47]. As the amplitude dynamics introduces an additional degree of complexity, it needs to be verified if the analytic predictions made for coupled limit-cycle oscillators [48] (valid for phase oscillators or supercritical Stuart-Landau oscillators) still hold. Selecting 40Hz as a typical frequency of gamma oscillations, we set all units with identical natural frequency to exclude additional effects of frequency dispersion [49,50], and perturb all units with uncorrelated white noise, considering that units resonate at their natural frequency in the presence of background noisy activity [51]. Assuming the generalizability of collective synchronization frequencies to delay-coupled damped oscillators, we hypothesise to identify a critical range of global model parameters (global coupling and conduction speed) where metastable synchronization generates the transient emergence of sub-gamma collective oscillations, approximating features of human MEG recordings.'

Text modified in the DIscussion:

'Specifically, we first demonstrate the generalizability of a synchronization mechanism described for networks of delay-coupled limit-cycle oscillators to networks of delay-coupled damped oscillators (i.e., in the subcritical range of a Hopf bifurcation). This is important for the neuroscience field, since empirical electrophysiological recordings show that local field oscillations in the gamma frequency band are not limit-cycle oscillations (as considered in previous models using the Kuramoto of coupled oscillators [20]), but instead emerge only transiently. Therefore, the substantial reduction of brain areas to phase oscillators in Cabral et al. (2014) has raised concerns on the generalizability of the proposed mechanism to more realistic settings, given the demonstrated importance of considering the amplitude dynamics on the connectivity between phases [48,61,62].
Subsequently, we extend on previous brain network modelling works by demonstrating that the synchronization frequency can be approximated analytically from global model parameters, namely the number of units, the mean coupling strength, the average time delay between units, and the mean natural frequency of the units. Regarding the latter, we show that the collective synchronization frequency is insensitive to the spread of frequencies across units, in line with theoretical predictions25 (Supplementary Figure S8).
These insights are crucial to explain the macroscopic spatiotemporally organized oscillatory signals detected with EEG/MEG at sub-gamma frequencies, without explicitly introducing these oscillations

in the model [63]. Here, we consider that only gamma-frequency oscillations can be generated at the local neuronal level, with power at other frequencies resulting purely from synchronization with time delays. Furthermore, we demonstrate the impact of global model parameters in the modulation of frequency-specific collective oscillations emerging across space and time. The detailed characterization of metastable oscillatory modes in terms of number of units synchronizing together, duration and occupancy provides a new framework to analyse collective brain oscillations complementary to frequency-specific envelope functional connectivity analysis.

Minor:
P18 L257 in SI, what are the state-of-the-art tractography algorithms? Please identify which tractography method was used to make the connectome in this study.

Authors' reply: Thank you for pointing this out. We now describe in detail the methods to estimate the connectivity and distance matrices used in the brain network model in the Methods section:

New Text in the Methods section:

'The NxN matrices of structural connectivity, C, and distances, D, used for the network model were derived from a probabilistic tractography-based normative connectome provided as part of the leadDBS toolbox (https://www.lead-dbs.org/) [83]. This normative connectome was generated from diffusion-weighted and T2-weighted Magnetic Resonance Imaging (MRI) from 32 healthy participants (mean age 31.5 years old ± 8.6, 14 females) from the Human Connectome Project (HCP). The diffusion-weighted MRI data was recorded for 89 minutes on a specially-designed MRI scanner with more powerful gradients then conventional scanners. The dataset and the acquisition protocol details are available in the Image & Data Archive under the HCP project (https://ida.loni.usc.edu/). DSI Studio (http://dsi-studio.labsolver.org) was used to implement a generalised q-sampling imaging algorithm to the diffusion data. A white-matter mask, derived from the segmentation of the T2-weighted anatomical images, was used to co-register the images to the b0 image of the diffusion data using the SPM12 toolbox (https://www.fil.ion.ucl.ac.uk/spm/software/spm12/). Within the white-matter mask, 200,000 most probable fibres were sampled for each participant. Then, fibres were transformed to the standard Montreal Neurological Institute (MNI) space applying a nonlinear deformation field derived from the T2-weighted images via a diffeomorphic registration algorithm [84]using the LeadDBS toolbox. The individual tractograms were then aggregated into a joint dataset in MNI standard space resulting in a normative tractogram representative of a healthy young adult population and made available in the leadDBS toolbox [83].

The NxN matrices were computed from the normative tractogram using the Automated Anatomical Labelling (AAL) parcellation scheme [85] with N=90 cortical and subcortical areas, by calculating the number of tracts, C(n,p), and mean tract length, D(n,p), between the voxels belonging to each pair of brain areas n and p. Details on the additional dataset and parcellation scheme used in Supplementary Material are reported in Supplementary Methods section V and Figure S14.'

Text added in the Supplementary Methods Section V, Structural Connectome:

'To evaluate the robustness of our model results to the choice of the parcellation scheme, the NxN matrices of structural connectivity, C, and distances, D, were obtained for a distinct parcellation

scheme considering N=200 brain areas (cortical only) defined by Schaefer and colleagues[75], applying the same methodology to the same normative connectome from 32 healthy HCP participants provided by the leadDBS toolbox (https://www.lead-dbs.org/)

In addition, we replicated the results using a distinct normative connectome available in the LeadDBS toolbox, derived from a neuroimaging dataset from 985 healthy participants also from the Human Connectome Project. In this dataset, diffusion-weighted MRI was recorded for 59 minutes using a multiband accelerated EPI pulse sequence on a conventional scanner. The acquisition protocol can be found under the "1200 HCP release" section (https://www.humanconnectome.org/). Briefly, probabilistic tractography was calculated for each participant with the leadDBS toolbox and a multispectral warp based on T1-weighted, T2-weighted and diffusion-weighted images (implemented via ANTs SyN registration (http://stnava.github.io/ANTs/)) was used to normalise the fibre tracts to the MNI standard space. Every participant had 6000 fibres sampled and aggregated to a joint dataset in MNI standard space resulting in a normative tractogram representative of a population of healthy humans.'

Reviewer #2:

The manuscript "Synchronization in the connectome: Metastable oscillatory modes emerge from interactions in the brain spacetime network" by Cabral et al. proposes a connection between transient brain rhythms and weakly stable synchronization of distant brain areas, emphasizing the role of signal transmission delays in the emergence of transient rhythms. To support their hypothesis, the authors present results of extensive numerical simulations carried out on a network of 90 Stuart-Landau oscillators, representing 90 brain areas, where connection strengths and conduction delays are derived from physical brain connectivity data.

The construction of the network based on real data is in itself a nice piece of work. Moreover, the simulations are extensive and contain rich results, some of which are already spelled out in the manuscript. The main findings indicate that conduction delays play a crucial role in shaping the frequency spectrum of coupled systems, and that synchronization under delays induces a shift to lower frequencies as well as a decrease in amplitude. Some of the observations are probably not completely surprising for theoretical scientists working with delay systems (as already referenced in the manuscript), but they are demonstrated here more concretely and quantitatively for a more realistic brain-like structure. It can be expected that the results provided here can provide even further inspiration for future studies in unraveling the details of brain oscillations, as well as in the wider area of complex networks.

Authors' reply: We thank the reviewer for recognizing the importance of bridging known concepts from the physics of delay-coupled systems to realistic brain-like structures to advance in the mechanistic understanding of brain oscillations. This was the main motivation of this work and we share the expectation that this will 'provide even further inspiration for future studies', extending beyond the neuroscience field to 'the wider area of complex networks'. As the reviewer will find, in this revised version of the manuscript we have improved in the clarity, novelty and robustness of our results and link more directly to MEG analysis in the final section. All changes in the manuscript are marked in blue font.

The manuscript is generally well-written, although a thorough reading and editing is recommended. There are some spelling errors (e.g. "conduction") and some consistency in typesetting notation (e.g. consider how differently the function "sin" is formatted in equations (3) and (4)). Finally I am not sure why the symbol "*" is used for multiplication in equations; it is not very standard (except in coding) and it is also not used consistently throughout the manuscript.

Authors' reply: We thank the reviewer for noting these errors, which have now been corrected. All co-authors have performed a thorough reading of the manuscript, and all corrections in the manuscript are marked with blue font.

Reviewer #3:

The authors use a brain network model to simulate brain dynamics and study spatio-temporal synchronization dynamics of brain regions. They break down oscillations into standard frequency bands studied in neuroscience and compare simulated results to that obtained from MEG data. They show that for certain ranges of regional delays and coupling strengths that the model can reproduce the slow frequency brain observations observed in real data. I fully support the use of brain network models to explore oscillations and synchrony in brain dynamics, but I feel that there is much analysis missing from the paper and that the main results are somewhat expected. This paper feels like a starting point for a more rich analysis that could link more directly to questions in neuroscience.

Authors' reply: We appreciate the reviewer's full support for 'the use of brain network models to explore oscillations and synchrony in brain dynamics' and we thank the reviewer for proposing additional analysis to complement our work and link more directly to questions in neuroscience. As the reviewer will find, in this revised version of the manuscript we now include new supplementary analysis to show the robustness of the results to changes in the connectome structure, in the frequency of the oscillators and in the parcellation scheme. Furthermore we demonstrate the adequacy of the integration step and include a new figure to illustrate the spatial configuration of the brain areas most involved in metastable oscillatory modes in each frequency band. All changes in the manuscript are marked in blue font. We are hopeful that these improvements to the paper fulfil the exacting standards required for publication in Communications Physics.
Regarding the reviewer's comment '*This paper feels like a starting point for a more rich analysis that could link more directly to questions in neuroscience.'* We share the enthusiasm and are hopeful this will be the case. We can advance that the model is already being used in new projects linking directly with source-projected MEG data and exploring the impacts of brain stimulation techniques. For the current paper, we chose to focus on the construct validity from a physics perspective of the hypothetical *mechanism* driving the collective brain oscillations - which remains unclear -, leaving the direct applications for subsequent and more neuroscience-oriented works.

Major general concerns:

1. It appears that the authors are building their structural matrix from the average connectome calculated from 32 individuals and then averaged over individuals. It has been shown that individual variability in the structural network used in these models leads to enhanced variability in the dynamics that they output. If the goal is to compare with individual MEG data, the structural networks used in the model need to be the structural networks from those same individuals. From what I can tell, no attempt was made to model separate dynamics at the individual level, and given that the MEG data is from 89 subjects, it is unclear if there is any overlap between the subjects used to generate structural and functional networks.

Authors' reply: We fully agree with the reviewer that individual variability in the structural network is a major contributor for variability across subjects, and there have been a number of works - including from our co-authors - showing how small differences in structural connectivity can impact the resulting dynamics (Cabral et al. 2012, Vasa et al., 2015). However, this variability in structural networks alone does not entirely explain the variability observed in the dynamics, in particular

because the same individual can exhibit very different activity patterns depending on the behavioural/physiological condition (e.g., wakeful rest vs sleep (Tagliazucchi et al., 2016) or wakeful rest vs anaesthesia (Barttfeld et al., 2015)), while the structural substrate remains static over the same time scales. And this is precisely the point we aimed to address with this analysis: that the same connectivity structure can give rise to different dynamic outputs depending on global parameters. Showing that the differences in MEG power spectra observed across subjects can be explained by small changes in the coupling strength or in the conduction velocity while keeping the same structural connectome demonstrates the importance of considering additional contributors to explain both between- and within-subject variability beyond structural variability alone.

Furthermore, although it has been shown that structural connectomes naturally vary across subjects, there is also an additional degree of variability induced by the methodology itself. In other words, structural connectomes constructed from scan-rescan diffusion MRI recordings from the same individual may exhibit similar variability as across individuals). This is often assessed by Inter-class Correlation (ICC) where between- and within-subject variability is computed. It has been shown, for example, that ICC is not uniform for the entire structural connectome and varies for different structural connections due to the probabilistic nature of tractography algortihms (Tsai 2018, Bonilha et al. 2015).

In that direction, normative structural connectomes serve as a good choice for mechanistic/phenomenological brain network models since they maximise the reliability of structural connections across subjects. In this work, normative connectomes derived as part of the leadDBS project were used. They were derived from the Human Connectome Project Database of 32 and from 985 individuals. We have rerun all our simulations using the structural connectome from 985 individuals showing that our results hold when using a different, yet still group normalised, structural connectome.

Following the reviewer's comment, we now explain more clearly the point we aimed to address with this analysis in the modified text in the Results section (copied below), provide more details on the methodology to obtain a normative connectome (from 32 individuals) and the MEG power spectra (89 non-overlapping individuals but with similar age range and gender ratio) in the Methods section (copied below), and report the results using a different normative connectome (985 individuals) in the Supplementary Information.

Barttfeld, P., Uhrig, L., Sitt, J. D., Sigman, M., Jarraya, B., & Dehaene, S. (2015). Signature of consciousness in the dynamics of resting-state brain activity. *Proceedings of the National Academy of Sciences*, *112*(3), 887-892.

Bonilha, L., Gleichgerrcht, E., Fridriksson, J., Rorden, C., Breedlove, J. L., Nesland, T., ... & Focke, N. K. (2015). Reproducibility of the structural brain connectome derived from diffusion tensor imaging. *PloS one*, *10*(9), e0135247.

Cabral, J., Hugues, E., Kringelbach, M. L., & Deco, G. (2012). Modeling the outcome of structural disconnection on resting-state functional connectivity. *Neuroimage*, *62*(3), 1342-1353.

Tagliazucchi, E., Crossley, N., Bullmore, E. T., & Laufs, H. (2016). Deep sleep divides the cortex into opposite modes of anatomical–functional coupling. *Brain Structure and Function*, *221*(8), 4221-4234.

Tsai, S. Y. (2018). Reproducibility of structural brain connectivity and network metrics using probabilistic diffusion tractography. *Scientific reports*, *8*(1), 1-12.

Váša, F., Shanahan, M., Hellyer, P. J., Scott, G., Cabral, J., & Leech, R. (2015). Effects of lesions on synchrony and metastability in cortical networks. *Neuroimage*, *118*, 456-467.

Modified text in the Results section:

"Given the observed (and well-established) variability between MEG power spectra across individuals (Figure 2a), we investigate the extent to which this variability can be associated with changes in global model parameters, while keeping the structural connectivity unchanged. To do so, we identify the pair of model parameters that approximates the individual MEG power spectra of each of the 89 participants, falling in 29 pairs of parameters (white asterisks in Figure 2b). Notably, this reveals a confined region in parameter space for a range of average delays of 2 to 11 milliseconds, with slight changes in the coupling strength and conduction speed maximizing the fit to individual MEG power spectra, while the structural connectivity remains unchanged. These results do not exclude the role of individual variability in structural connectivity across subjects but reveal additional parameters that modulate a network's frequency spectrum. This serves to demonstrate that the same connectome structure can support distinct activity patterns depending on global model parameters, with longer/shorter time delays and stronger/weaker coupling inducing shifts in the peak frequency and modulating the distribution of power across the spectrum (Figure 2b)."

Modified text in the Methods section:

"This normative connectome was generated from diffusion-weighted and T2-weighted Magnetic Resonance Imaging (MRI) from 32 healthy participants (mean age 31.5 years old ± 8.6, 14 females) from the HCP. [...] Within the white-matter mask, 200,000 most probable fibres were sampled for each participant. Then, fibres were transformed to the standard Montreal Neurological Institute (MNI) space applying a nonlinear deformation field derived from the T2-weighted images via a diffeomorphic registration algorithm [84]. The individual tractograms were then aggregated into a joint dataset in MNI standard space resulting in a normative tractogram representative of a healthy young adult population and made available in the leadDBS toolbox [83].

[...]

The power spectra from human resting-state MEG signals were also downloaded from the Human connectome Project (HCP) database [...]. The MEG power spectra are provided for 89 healthy participants at rest (mean age 28.7 years old, range 22–35, 41 female) distinct from the 32 participants from which the structural connectomes were derived, but with similar age range and gender ratio.

2. Given the above, I don't understand the motivation to even attempt to look at how the model dynamics align with individual MEG power spectrums. I wouldn't expect the model to match well with across subjects given that the underlying structural matrix isn't subject-specific.

Authors' reply: We understand the reviewer's concern and we now better justify our rationale. As the reviewer noted above, it is generally assumed that individual variability observed in brain activity is inherently associated with structural variability. Yet, although there is no doubt that structural connectivity shapes network dynamics, there are additional elements that influence the activity patterns of a networked system, which can explain the transition between regimes with distinct dynamical features without a necessary change in the connectivity structure.

Here, we aimed at demonstrating that the same connectome structure can exhibit distinct activity patterns with power spectra depending on model parameters. Moreover, we focused on demonstrating that, for a bounded range of parameters, the networked system can display oscillatory activity with a spectrum of frequencies qualitatively similar to the one detected in M/EEG, even if these oscillations are not explicitly introduced in the model equations. To do so, we first used the average MEG power spectrum (across all sensors and all individuals) to find the range of parameters where the disparity between the model and empirical data was minimal. But given the observed variability between power spectra across individuals, we found it very relevant to report that the variability in individual power spectra could be associated with small changes in global parameters, such as the coupling strength between brain areas. Of course this does not exclude the role of individual changes in structural connectivity, but introduces additional parameters that control the oscillatory activity, which may be easier to modulate than structural connections.
These results provide a valuable insight on the influences of global model parameters to the subject-specific changes of the power-spectrum when the underlying structural connectivity remains fixed.

3. The authors discuss spatial synchronization, but seem to be looking only at things such as the number of units recruited into MOMs and the frequency specific envelope covariance matrices. For this to be interesting to a neuroscientist, one wants to know which brain regions are recruited into the oscillations. Do those regions relate to known cognitive or functional communities? One expects there to be metastable communities that synchronize at different frequency bands. Which brain regions are involved in these patterns is the interesting part.

Author's reply: We thank the reviewer for raising this crucial point and we have restructured the final section of the Results, now entitled *'Frequency-specific functional connectivity'* in order to link more closely the model results to MEG analysis methods. In particular we now focus on the functional connectivity estimated as the correlations between the amplitude envelopes of band-pass filtered oscillations, as commonly applied in source-reconstructed MEG data. We now report these in the new Figure 6.

New Results section:
'Frequency-specific functional connectivity

To link with studies of functional connectivity in MEG, we further investigate how the model parameters modulate the correlation between the amplitude envelopes across frequency bands. To do so, we band-pass filter the signals in each frequency band, extract the amplitude of the Hilbert transform and report the envelope correlation matrices in Figure 6 for each frequency band and for four representative sets of model parameters. For weak coupling, the envelope correlations are close to zero (Pearson's correlation coefficient cc<0.1 for all pairs of brain areas), indicating that the coupling is insufficient to drive meaningful functional connections between brain areas. For global parameters in the optimal range (here K=10 and MD=3ms), different brain areas exhibit correlated envelopes, with stronger correlations (up to cc=0.78) being detected in the alpha frequency range. In contrast, for strong coupling the functional connectivity in the alpha is reduced down to a maximum pairwise correlation of cc=0.25, while in turn the envelopes of delta and theta oscillations are strongly correlated across the brain (up to cc=0.89). Keeping the optimal range of global coupling, K=10, but increasing the delays to an average of MD=20ms, envelope functional connectivity is detected mostly

in the delta frequency range. This illustrates how, given the same underlying spacetime network structure (i.e., the matrices of coupling weights C and distances D), changes in global parameters strongly affect the envelope functional connectivity patterns at different frequency bands.

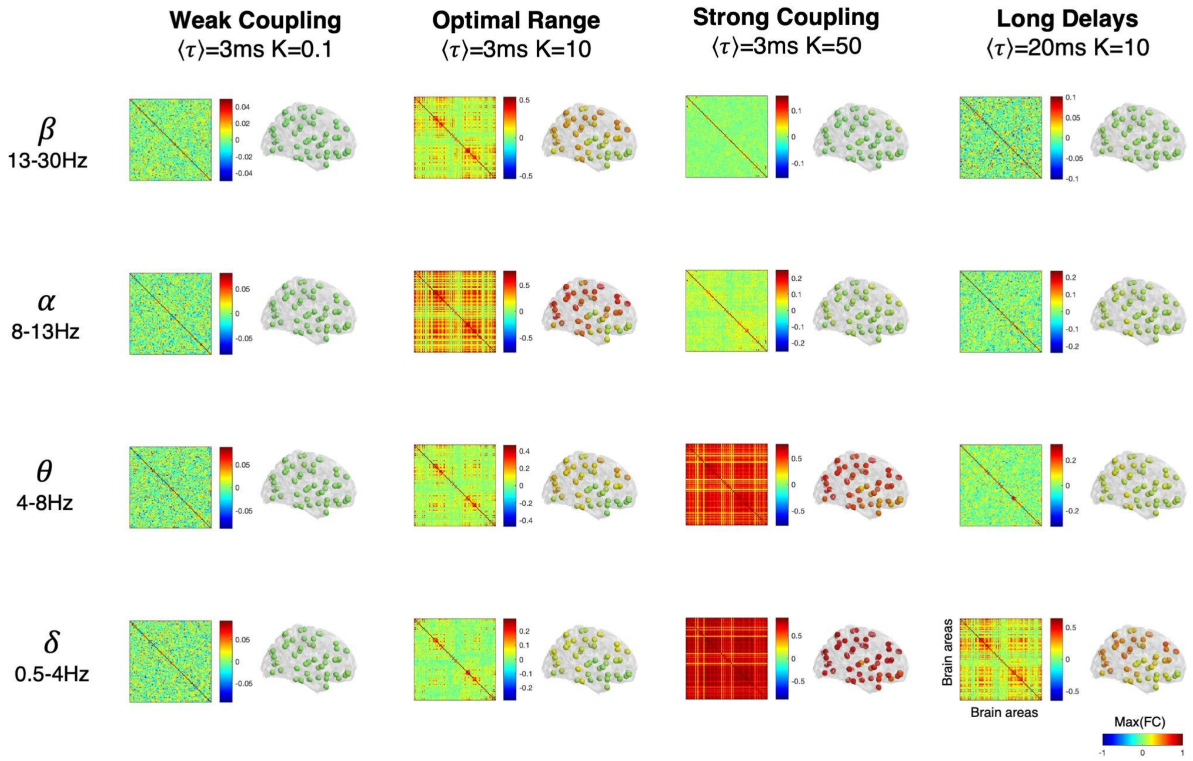

Figure 6. Influence of global model parameters in frequency-specific envelope functional connectivity patterns. For different sets of model parameters, we report the frequency-specific envelope correlation matrices (colormap limits are scaled by the maximum absolute correlation and centred at zero). Next to each matrix, each of the N=90 brain areas is represented as a sphere placed in its centre of gravity and coloured according to the maximum envelope correlation to any other brain area.

To illustrate the level of functional connectivity across the brain, next to each correlation matrix in Figure 6, we represent each area as a sphere placed at its centre of gravity and coloured according to the strongest correlation with any other brain area. This shows that, for the optimal range of parameters, the areas exhibiting the strongest functional connectivity in the alpha band are distributed mostly in posterior and dorsal cortical areas, aligning with empirical observations of stronger functional connectivity in the alpha band in the visual and somatomotor systems.'

More specific comments:

1. The analysis was performed on the 90 region AAL atlas. Do the results hold for other parcellations with a larger number of regions?

Authors reply: We agree with the reviewer on the importance of demonstrating the generalizability of the mechanism. Following the reviewer's suggestion, we have rerun all the simulations using instead the Schaefer parcellation with 200 brain areas (see Supplementary Methods, section V, Figure S14) . This parcellation differs from the previous parcellation not only in terms of the number

of regions, but also because it only covers the cortex, whereas the AAL includes both cortical and subcortical areas. Although the results remain qualitatively similar (revealing the emergence of metastable oscillatory modes with similar spatial distribution across the cortex) it is important to note that the brain regions forming the metastable communities are strongly dependent on the specific resolution and topology of the connectome structure (i.e., modularity) and on the brain parts included (i.e. cortical, subcortical). We added these results in the Supplementary Methods, Section V, Figure S14-15-16-17 and added new text in the Results and in the Discussion sections.

Text added in the Results section:
'However, it is important to consider that the specific spatial configuration of functional connections is inherently dependent on the resolution and topology of the structural connectome, which is known to depend on the parcellation scheme and on the brain parts (i.e. cortical, subcortical) considered . In Supplementary section V, we report the same results using a structural connectome including 200 cortical-only brain areas [54]. These results suggest that the phenomenology of MOMs is robust to changes in the parcellation scheme (Supplementary Figures S16 and S17). Most importantly, this analysis illustrates how frequency-specific functional connectivity patterns depend sensitively on global variables modulating the distributed dynamics, while the structural connectivity remains unchanged.'

2. I understand the desire to look at the dependence on coupling strength and delays, but given that much other work looking at coupled oscillators outside of neuroscience applications also has investigated this, an interesting and relevant parameter to explore in this case would be the natural frequency of the oscillators. From what I can tell, all oscillators were set to a natural frequency of 40 Hz. Are the results robust to different choices? I would expect that different brain regions oscillate at different natural frequencies, so exploring how the model depends on this parameter choice could be a large contribution to linking coupled oscillator theory to brain dynamics.

Author's reply: We acknowledge the importance of studying the effect of the spread of natural frequencies, in order to align with theoretical studies in coupled oscillators with frequency dispersion. We have included a new figure in the Supplementary Methods, Section II, Figure S8, where we show the robustness of our results to the spread of natural frequencies (i.e., keeping the mean at 40Hz and increasing the standard deviation up to 20Hz). This is aligned with the commentary in the Discussion of Niebur and colleagues (1991) who wrote:
"Our analysis has shown that these phenomena depend only on the generic periodicity of the interaction between the phases and that they are relatively insensitive to the spectrum of intrinsic frequencies."
Since here we aimed to demonstrate that the emergence of novel frequencies in the spectrum where associated to the connectome spacetime structure and *not* to the spread of natural frequencies, we chose to use identical oscillators, in order to demonstrate that complex oscillatory dynamics similar to what is observed empirically can emerge from identical oscillators.

Text added in the Introduction section:
'Selecting 40Hz as a typical frequency of gamma oscillations, we set all units with identical natural frequency to exclude additional effects of frequency dispersion (Strogatz and Mirollo, 1991; Petkoski et al., 2013).

Text added in the Results section:

'The robustness of this prediction to distributed natural frequencies is reported in Supplementary Figure S8.'

Text added in the Discussion section:

'While the investigation of mechanistic principles and control parameters benefits from reduced complexity, adding heterogeneity is certainly needed to improve the fitting to real brain activity from individuals in different conditions. Building up on these fundamental aspects, additional degrees of complexity can be added to the model, namely by considering more fine-grained connectome structures, considering non-homogeneous intrinsic frequencies and damping properties, or even replacing the noisy input by dynamic concentration patterns to mimic local neuromodulatory effects. Further, given the potential generalizability of this synchronization mechanism, we expect our analysis may provide valuable insight to interpret some of the complex self-organizing phenomena emerging in more realistic biophysical models of neural networks [67,68] for which a precise analytic prediction cannot be solved.'

Niebur, E., Schuster, H.G. and Kammen, D.M., 1991. Collective frequencies and metastability in networks of limit-cycle oscillators with time delay. Physical review letters, 67(20), p.2753.

Petkoski, S., Iatsenko, D., Basnarkov, L. & Stefanovska, A. Mean-field and mean-ensemble frequencies of a system of coupled oscillators. *Physical Review E* **87**, 032908 (2013).

Strogatz, S. H. & Mirollo, R. E. Stability of incoherence in a population of coupled oscillators. *Journal of Statistical Physics* **63**, 613-635 (1991).

3. Line 260-262: "Importantly we note that for the model to exhibit the predicted behavior, the integration step for numerical simulations needs to be sufficiently small…". This is a concerning statement. Of course one much choose an integration step small enough that the results are stable – this is just related to correctly performing numerical integration. If the results are dependent upon the integration time step, then the numerical integration is not being performed correctly. Since the authors are injecting noise into the equations, it might be necessary to perform stochastic numerical integration – although it is unclear if this was done.

Author's reply: We fully agree with the reviewer that 'one must choose an integration step small enough that the results are stable' and this is precisely what we aimed to explain with our sentence, although this was not very clear. We did verify the adequacy of the step for numerical integration by ensuring the stability of the results when reducing the order of magnitude of the integration step from 1e-4, 1e-5 to 1e-6 seconds. As we now show in Section VI, Figure S18-19-20, the simulations with dt = 1e-6 seconds are qualitatively similar. We wanted to make this point clear because the numerical integration of such a large system of stochastic delay differential equations needs to be very precise, and, although alternative algorithms exists to solve DDEs, we found that an Euler scheme with sufficiently small time step (and saving only a running history at this time resolution) was, to our experience, the most cost-efficient way to obtain stable results that matched analytic prediction.

Regarding the added noise, we did perform stochastic numerical integration by multiplying the noise standard deviation by the square root of the integration step, and we now explicitly state this in the corresponding Methods section: ‘*In this analysis, the system is perturbed with uncorrelated white noise, where η1 and η2 are independently drawn from a Gaussian distribution with mean zero and standard deviation β=0.001* ***(integrated as β*√dt)***.’

Text corrected in the Discussion section:

‘We note that for the numerical integration of stochastic delay differential equations to be stable and align with analytic predictions, the time step for numerical integration needs to be sufficiently small and a running history needs to be saved for the length of the maximum delay between units, which significantly increases the computation times when compared to simulations where delays are neglected (here the numerical results were found to stabilize for dt 10-4 seconds, see Supplementary Figures S18-S20).’’

Author’s comment: We wish to thank the reviewer for the suggestions to improve the clarity and reinforce the validity of our manuscript.

REVIEWERS' COMMENTS:

Reviewer #1 (Remarks to the Author):

The authors well addressed my comments. This manuscript is now ready to be published.

Reviewer #2 (Remarks to the Author):

The authors have clarified several significant points and improved the presentation in their revised manuscript, which I believe is suitable for publication in Communications Physics.

Reviewer #3 (Remarks to the Author):

I appreciate the work that the authors have done to clarify their position on looking at variability due to parameters different from structural variability. I am happy with the changes made but would like to note that when comparing Figures 6 and S17, the spatial specificity in the alpha band disappears for the atlas with more brain regions, so the statement: "In Supplementary section V, we report the same results using a structural connectome including 200 cortical-only brain areas. These results suggest that the phenomenology of MOMs is robust to changes in the parcellation scheme (Supplementary Figures S16 and S17)." should be modified slightly.

Response to Referees

Reviewer #1

The authors well addressed my comments. This manuscript is now ready to be published.

Reviewer #2

The authors have clarified several significant points and improved the presentation in their revised manuscript, which I believe is suitable for publication in Communications Physics.

Reviewer #3

I appreciate the work that the authors have done to clarify their position on looking at variability due to parameters different from structural variability. I am happy with the changes made but would like to note that when comparing Figures 6 and S17, the spatial specificity in the alpha band disappears for the atlas with more brain regions, so the statement: "In Supplementary section V, we report the same results using a structural connectome including 200 cortical-only brain areas. These results suggest that the phenomenology of MOMs is robust to changes in the parcellation scheme (Supplementary Figures S16 and S17)." should be modified slightly.

Authors: We thank the reviewer for the positive comments. Following the reviewer's comment, we have now modified the text in page 10 to:

'In Supplementary Methods 1, we perform the same analysis on data simulated using a structural connectome including 200 cortical-only brain areas[54]. These results show that, while the phenomenology of MOMs is robust to changes in the parcellation scheme, the spatial specificity across frequency bands is sensitive to the parcellation scheme considered (Supplementary Figures 14-17).'
